# Precision Medicine for Intracranial Atherosclerotic Disease

**DOI:** 10.3389/fneur.2021.646734

**Published:** 2021-02-10

**Authors:** David S. Liebeskind

**Affiliations:** Department of Neurology, Neurovascular Imaging Research Core & UCLA Stroke Center, University of California, Los Angeles, Los Angeles, CA, United States

**Keywords:** intracranial atherosclerosis, stroke, hemodynamic, precision medicine, imaging

## Abstract

Diagnostic and therapeutic strategies for intracranial atherosclerotic disease (ICAD) have vastly expanded within the last several years. Challenges and concrete initiatives have emerged in the implementation of precision medicine for ICAD, focusing personalized treatment for the prevention of stroke and cognitive impairment around pathophysiology. Theranostics for ICAD incorporates an integrated diagnostic and therapeutic approach tailored to a specific individual. The ICAS 2019 meeting provided a roadmap for accelerating global innovation, underscoring the epidemiology, prior scientific evidence from trials, diagnostic tools or imaging, novel biomarkers, management approaches, and a broad range of treatments including many new medications, endovascular, and surgical strategies. This thematic overview provides perspective on current definitions for arterial stenosis, symptomatic lesions and outcomes or endpoints in clinical trials. Imaging correlates are reviewed, from routine multimodal CT or MRI to advanced angiographic techniques. The temporal features of ICAD and longitudinal observation are considered with respect to management and risk factor modification. The evolving science of multivariable interactions in ICAD and use of big data are explored, followed by an overview of recently launched clinical trials.

## Introduction

Recurrent stroke due to intracranial atherosclerotic disease (ICAD), the leading cause of stroke worldwide, causes an overwhelming burden of disability (1, 2). As the most common etiology of ischemic stroke, effective treatment strategies for acute ischemia and secondary stroke prevention are greatly needed. The neurological impact of ICAD is likely vastly underestimated by the incidence of recurrent stroke, as relatively mild clinical severity, involvement of secondary or less apparent functional regions of the brain and lack of continuity of care decrease reported recurrent events. Furthermore, recurrent ischemia may not cause clinical stroke events, yet cognitive and other neurological impairment may ensue (3). Despite this public health priority, there remain no proven treatments for acute or recurrent ischemia due to ICAD (4). Brain or cerebrovascular health is a relatively novel concept, unlike the established prominence of cardiovascular health. Given the prevalence and impact of ICAD on neurological disability, developing effective treatment for ICAD represents a key goal in sustaining brain health. The preponderance of “silent” ischemia without overt clinical stroke syndromes makes the use of imaging surveillance more important. More detailed clinical and imaging evaluation of individuals with ICAD is therefore necessary to properly tackle this disease.

Precision medicine for ICAD may leverage the extensive data that exist from routine imaging and clinical data (5–9). Although precision medicine in other disorders evolved from genomics, such phenotypic data may provide exquisite delineation of individual risk and disease trajectory. Advancing the field of ICAD at the population level may be fueled by understanding individual patient management with a focus on granular details. Theranostics of precision medicine entails a combination of diagnostic and therapeutic considerations, tailoring one to the other, based on specific details of a given patient. Even more so, such details are focused on the exact clinical presentation and timecourse, whether acute or chronic. The goal of such an approach is to provide the right treatment, at the right dose or use, for the right patient, at the right time. Such an approach is conceptually the converse of the data provided by a randomized, controlled trial of ICAD. Rather than controlling for individual distinctions in myriad variables from subject to subject and looking solely at class-level response to the investigational treatment, precision medicine studies of ICAD would focus first on the extensive variables that distinguish one individual from the next.

Trials of ICAD have demarcated the landscape of stroke prevention in this subtype, delineating few conclusions about what strategies are most effective. The Warfarin-Aspirin Symptomatic Intracranial Disease (WASID) trial compared aspirin to warfarin, leaving no clear answer (2). The subsequent Stenting and Aggressive Medical Management for Preventing Recurrent Stroke in Intracranial Stenosis (SAMMPRIS) trial tested the role of angioplasty and stenting with dual anti-platelet therapy (DAPT) compared to DAPT alone (10). SAMMPRIS demonstrated an unfavorable risk of early stroke after stenting, yet the annual risk of stroke in these cases with >70% stenosis was still considerable. After SAMMPRIS in 2011, blanket statements about ICAD treatment followed, citing a “failure” of stenting and “best medical therapy” as 90 days of DAPT after an index ischemic stroke or TIA. For the last decade since SAMMPRIS, it has been difficult to study ICAD. ICAD studies have many facets that are relatively more complex than trials in acute ischemic stroke. ICAD is difficult to diagnose underlying complete occlusion of a proximal artery, the extent of acute ischemia is relatively modest and the longer-term outcomes of prevention studies are more challenging. The seminal ICAD trials, however, provided a wealth of data for important subgroup analyses.

## Intracranial Atherosclerosis 2019

Despite almost a decade elapsing since the last largescale therapeutic trial in ICAD, the research community has maintained interest in tackling this influential cause of stroke. Last year, the ICAS 2019 meeting provided a roadmap for accelerating global innovation, underscoring the epidemiology, prior scientific evidence from trials, diagnostic tools or imaging, novel biomarkers, management approaches, and a broad range of treatments including many new medications, endovascular and surgical strategies (11). At ICAS 2019, the global epidemiology of ICAD, including subclinical disease was assessed. Cognitive impairment due to ICAD was discussed and a variety of novel imaging diagnostics were scrutinized, ranging from ultrasonography to vessel wall imaging and computational fluid dynamics (CFD). Recent trials were reviewed including statins, PCSK9 inhibitors and a variety of new anti-platelet therapies. Endovascular and surgical studies were reviewed, underscoring promising future trial designs. Genetic factors and the role of precision medicine in ICAD were also debated. Despite this extensive list of potential avenues for future investigation, several limitations exist in trial design (6, 7).

## Targets and Definitions

Precise definitions and therapeutic targets are needed to properly study ICAD, yet numerous hurdles remain in how we define and characterize ICAD. Almost the entire field of ICAD has defined the disease substrate and therapeutic target as a focal stenosis of a proximal artery in the brain (4). Focal stenosis or luminal narrowing across a discrete arterial segment is only one manifestation of underlying atherosclerosis. ICAD has been equated with focal stenosis as it is easiest to identify, yet paradoxically the obsession with exact percentage of luminal stenosis has little significance when other variables such as hemodynamics are considered. Other imaging features such as perfusion in the downstream arterial territory or collateral status have rarely been considered. Indications for endovascular therapy remain ill-defined, as well. Although we colloquially refer to “best medical therapy,” it remains unclear which anti-platelet therapy combination should be used, for how long, and how we should tailor statins or PCSK9 inhibitors. Perhaps the greatest quandary relates to optimal management of blood pressure: what are target goals; what treatments are indicated; and how these factors relate to actual blood pressure response. Finally, it remains unclear whether endpoints should solely revolve around recurrent ischemic stroke or whether we should consider cognitive trajectories.

The designation of symptomatic vs. asymptomatic lesions in ICAD has been variably defined. Although strict definitions have been applied in randomized, controlled trials where an index stroke or TIA must be accompanied by focal neurological symptoms referrable to the downstream arterial territory, such strict definitions are rarely adjudicated. Furthermore, imaging evidence of ischemia in the downstream territory is seldomly used to define symptomatic arterial lesions.

**Figure 1:**
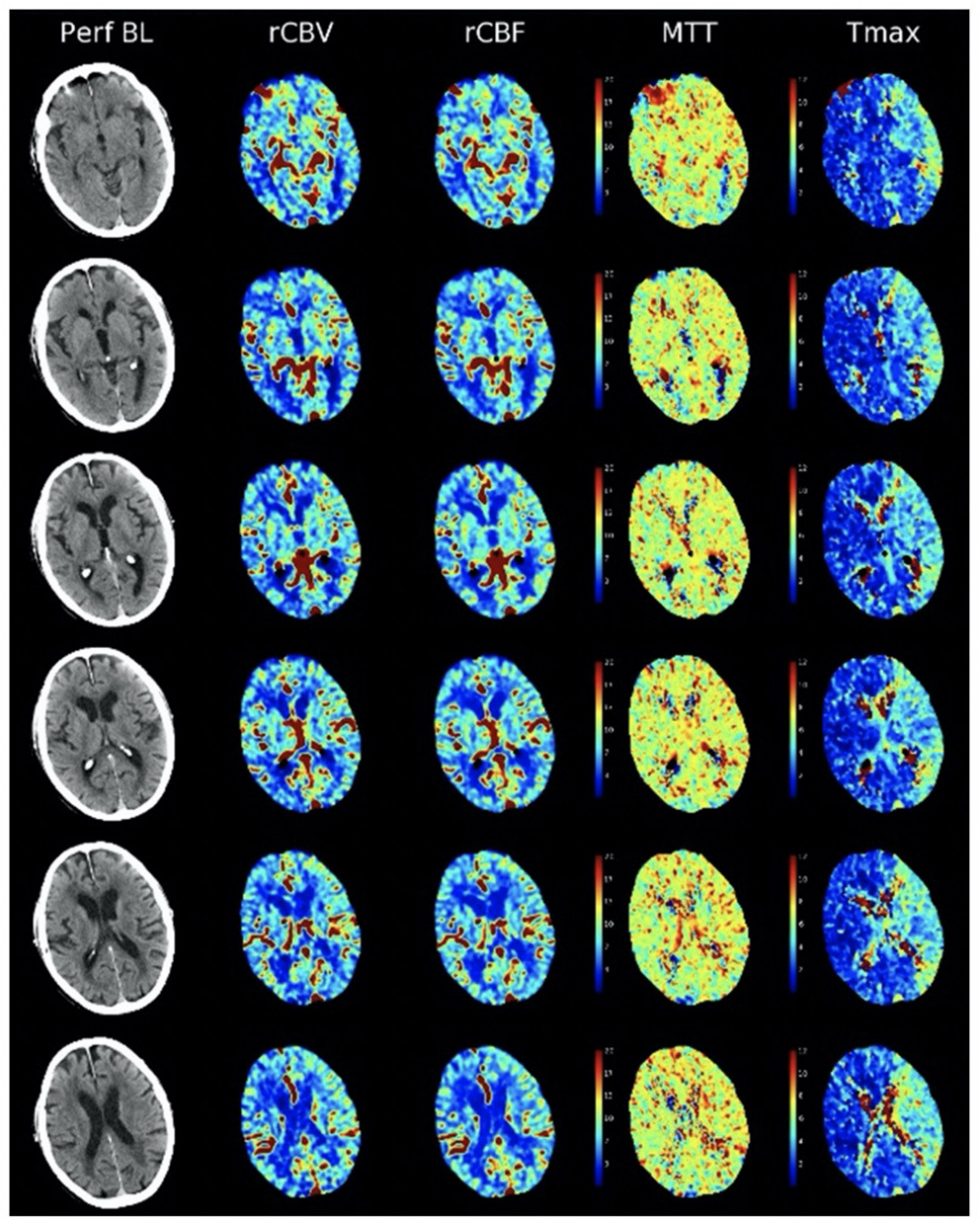
Non-contrast CT and CT perfusion maps of ICAD in the proximal left middle cerebral artery. A small, subcortical stroke is seen on non-contrast CT (left column, Perf BL). CT perfusion (right columns) reveals residual antegrade flow across the stenotic lesion, with preserved blood volume (rCBV), blood flow (rCBF), and mean transit time (MTT) with extensive areas of Tmax delays <6 s (Tmax).

Imaging definitions for ICAD have not been validated (12). On CT or MRI perfusion studies, definitions of infarct core and penumbra have been appropriated from acute ischemic stroke, without validation. For example, perfusion delays measured by time-to-peak or Tmax are radically different in ICAD when residual antegrade flow is present, compared to complete occlusion in acute ischemic stroke where the delays reflect several additional seconds of delayed arrival due to retrograde pial collateral flow. In ICAD with residual antegrade flow across a stenotic lesion, milder Tmax and other perfusion delays carry distinct significance (Figure 1). Arterial stenoses are typically diagnosed only when there is extreme narrowing of the arterial lumen, as most lesions below 70% stenosis are considered “mild” without consideration of downstream consequences. Even when downstream ischemia or perfusion delays are manifest, many lesions are designated as “mild.”

Our clinical definition of symptomatic ICAD lesions has been narrowed to index stroke events, as classification of TIAs often remains unclear. Clinical status is highly dependent on reporting of clinical events, leaving many gaps in management of patients with ICAD. Serial or continued evaluation of patients with ICAD is relatively uncommon, as we rely on patients reporting recurrent symptoms and we rarely assess sequelae, such as cognitive dysfunction, beyond symptomatic clinical complaints. In the overwhelming majority of cases, serial imaging evaluation is rarely pursued to detect recurrent ischemia.

## Beyond Focal Stenosis

Focal arterial stenosis is simply one subtype of ICAD, as a subset of a systemic atherosclerotic disorder. It reflects a particular manifestation of ICAD, much as coronary stenoses are a subtype of atherosclerotic coronary artery disease (CAD). Perhaps not surprisingly, many datasets have demonstrated a correlation between the presence of ICAD and co-existent CAD. When diffuse ICAD is present, it may be difficult to discern a focal arterial stenosis as the entire arterial segment may have concentric narrowing (13). As a result, even “mild” lesions of <70% stenoses may be difficult to diagnose, despite the presence of overt ICAD. Similarly, other subtypes of ICAD are neglected, such as dolichoectasia and intracranial calcifications that do not encroach on the arterial lumen. Dolichoectasia in the proximal arterial segments of the intracranial circulation may result from positive or outward remodeling of the arterial lumen due to atherosclerosis. Similarly, intracranial arterial calcifications may be apparent in the carotid siphon or distal vertebral arteries, reflecting a specific flow-related disease manifestation, distinct from focal stenosis. In sum, the resolute definition of ICAD as focal stenosis is likely overzealous, limiting our insight on the broader spectrum of atherosclerosis in the brain.

## Hemodynamics

**Figure 2:**
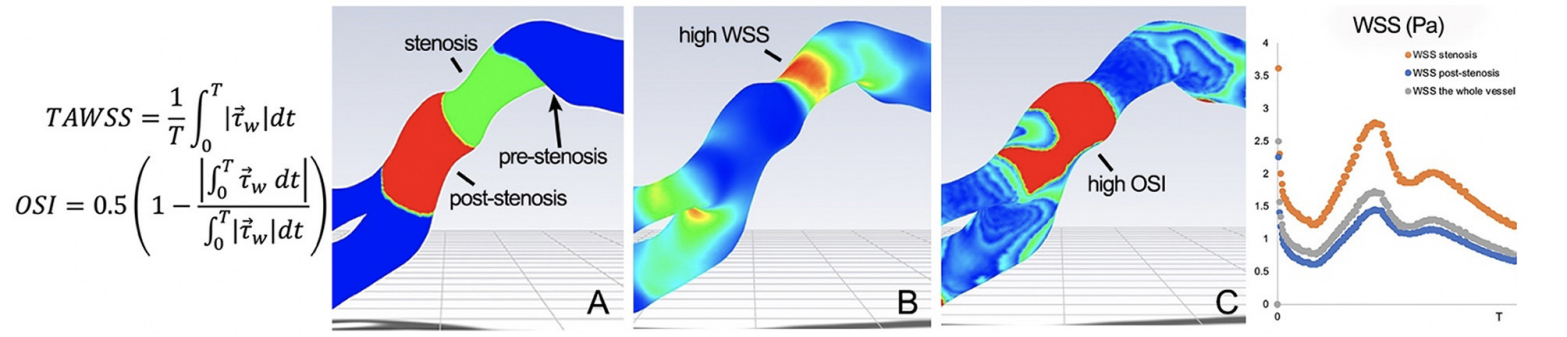
Pulsatile CTA CFD of MCA ICAD, revealing **(A)** a 4.9 mm stenotic segment (green) and corresponding 4.9 mm post-stenotic segment (red). The WSS map **(B)** depicts high TAWSS and WSSR in the stenotic segment. The OSI map **(C)** shows mean OSI of 0.31 in the post-stenotic segment. ${\overset{\to }{\tau }}_{w}$ represents the WSS vector and T represents the period of the cardiac cycle.

The hemodynamic impact of arterial lesions due to ICAD may be quantified by flow aberrations at the site of the arterial lesion or by blood flow delivery in the downstream arterial territory. ICAD stenoses of varying degree or percentage narrowing of the artery result in different effects on blood flow across the lesion and in the downstream bed. At the site of stenosis, high wall shear stress is often noted with post-stenotic vortices, low shear stress zones associated with prothrombotic, pro-inflammatory stimuli and oscillatory shear indices that may promote platelet aggregation (Figure 2) (14). In WASID, downstream patterns of residual antegrade blood flow and collateral circulation were shown to be an important predictor of subsequent stroke in the territory (15, 16). These blood flow patterns were more important predictors of recurrent stroke than the degree of narrowing. In SAMMPRIS (ICAD >70%), conventional angiography of collaterals was available in 376 subjects, revealing decreased antegrade flow in 50% and complete collateral compensation in 31% (10). More robust collaterals were noted in younger subjects, those with higher HDL, those participating in moderate exercise and non-smokers (all *p* < 0.05). Confirming the findings of WASID, more robust collaterals in SAMMPRIS were associated with markedly reduced rates of recurrent stroke.

## Counting Strokes—Acute-on-Chronic Ischemia

The nature of ICAD as an acute-on-chronic condition defies traditional approaches to trial design where it is assumed that the index hospitalization reflects the first stroke associated with the arterial stenosis. In a subset of cases, individuals presenting initially with symptomatic ICAD have no imaging evidence of prior infarction, however, many others have underlying evidence of prior strokes in the territory. MRI of acute ischemic stroke due to ICAD often shows relatively small DWI lesions (~1 cc) yet FLAIR may reveal much more extensive chronic ischemic lesions in the territory (17). Clinical definitions of stroke are applied, counting only events where focal symptoms have been ascribed to a discrete episode. As a result, ICAD patients may have had a varying degree of prior strokes in the territory, yet trials have neglected to count such strokes. A history of “old stroke” has been associated with worse prognosis. After SAMMPRIS, the U.S. Food and Drug Administration (FDA) warned that stenting should only be considered after 2 strokes, when a subject is at imminent risk of a third stroke (18). Counting such strokes, however, is therefore quite vague without detailed clinical and imaging data over time. These findings suggest that it is important to ascertain the temporal profile or history of disease with serial imaging and clinical data over time. In clinical practice, however, the lack of proven medical or endovascular treatments has been interpreted by some as an unproven need to follow ICAD patients over time.

## Myriad Mechanisms and Multiple Variables

The complexity of ICAD is rooted in the myriad mechanisms of ischemic stroke and the extensive list of variables that may affect such outcomes. Several biological mechanisms of stroke in ICAD have been invoked, including residual flow across the stenosis, decreased compensatory collateral perfusion, impaired vasomotor reactivity (VMR), plaque growth, distal emboli and perforator occlusion. Each of these mechanisms may be demonstrated with neuroimaging modalities, as in the MyRIAD prospective, observational study, yet interactions between these factors and the role of independent mechanisms remains an open question (17, 19). Even when using detailed imaging of ICAD, the cutpoints or thresholds for disease severity remain unclear, much as with percent arterial stenosis. For instance, it remains unclear what should be considered abnormal or deleterious with respect to blood flow velocities, fractional flow measures, Tmax perfusion thresholds, degree of VMR impairment, or number of emboli. Combining these imaging definitions with numerous other clinical, serological, or even genomic variables markedly increases complexity, yet likely improves prediction of disease course.

## Endpoints and Failure in Stroke Prevention

Defining the failure of stroke prevention or what constitutes an endpoint in studies of ICAD remains unanswered. Similar to controversy regarding the inclusion of TIA with acute stroke as an index event for entry into ICAD trials, the role of TIA as an endpoint is unclear. Recurrent TIAs may be difficult to adjudicate and may not carry the same clinical significance. It may be argued that recurrent TIAs without infarction may promote ischemic conditioning and impart better prognosis, overall. When stroke is used as an endpoint, the relationship to the parent artery stenosis is key. Stroke in the territory, rather than other stroke subtypes must be evaluated. The nature of each stroke, with respect to neurological deficits or severity and subsequent disability, should also be defined. Cognitive impairment has rarely been the focus of endpoints in ICAD studies. Interestingly, ~55% of subjects in SAMMPRIS had decreased cognitive performance, measured by the Montreal Cognitive Assessment (MOCA) (3). In ICAD, such cognitive dysfunction has been linked with impaired perfusion and the likelihood of recurrent stroke. These measures of failed stroke prevention must be qualified, however, as they relate to particular therapies or interventions and whether recurrent strokes may have been diminished rather than averted altogether. Recurrent stroke often prompts clinicians to switch a variety of anti-thrombotic strategies, without proven failure of such approaches compared with other therapies. Similarly, restenosis after stenting is often seen as a failure that prompts repeat endovascular therapy even when clinical symptoms may be inapparent.

## Big Data in ICAD

The extent of potential informative data in ICAD, including a wide range of clinical, imaging and other variables over time is seemingly boundless (6, 7, 20–22). The seminal WASID and SAMMPRIS trials incorporated voluminous amounts of data, even without detailed imaging variables explored in MyRIAD. Genomics of ICAD and serological measures remain largely unexplored. Only recently have any studies begun to explore the role of resistance or pharmacologic response to anti-platelet therapies. Blood pressure management has rarely been studied and physiological data on blood pressure variability or continuous measures over time is untouched. ICAD patients often keep blood pressure diaries, yet such data is often uncaptured. The volume, depth and dimensions of potential big data in ICAD is daunting yet carry the potential to serve as the basis for precision medicine approaches, delineating the optimal treatment of individual patients. Fortunately, monitoring of blood pressure, physical activity, and even cognition may now be tracked with mobile technologies. Data management in ICAD will undoubtedly be important in future studies. The incremental value of each variable is often questioned, yet this remains unknown at the outset. Observational studies of ICAD will play an important role, missing data is inevitable and advanced analytic approaches that consider an array of variables and clustering of data will be important to discern subtle, yet important interactions. Longitudinal evaluation and follow-up data are imperative. Selection biases must be accounted for and survival bias must be avoided when analyzing endpoint or long-term outcomes. Capturing data on the largest population of ICAD is most important for generalizability. These approaches should capture both the acute and chronic phases of ICAD disease course. Although the annual recurrent stroke risk of ICAD is relatively high, the majority of ICAD patients may remain stable over the course of 1 year. Ascertaining the serial trajectory of ICAD is a key priority for future research. The role of changes in neurological status or imaging features at two timepoints will be important in predicting the subsequent course, as well.

## Discussion of Emerging Studies and Future Trials in ICAD

The issues, gaps and corresponding opportunities for precision medicine in ICAD leave many approaches available for future research. Parallel approaches, incorporating observational studies and innovative interventional trial designs will likely emerge. Treatment or interventional studies may leverage novel methods such as platform trials (23). Both research strategies will require detailed and intensive data methodology for precision medicine insight. Such methods must entail relatively easy or practical data collection, including non-invasive, serial, and angiographic imaging studies. Available technologies may be leveraged to capture serology, genomics, routine imaging, blood pressure, physical activity and cognitive data. Infrastructure for such studies may build upon existing frameworks developed in recent studies like MyRIAD (17).

Ongoing imaging studies such as MyRIAD will be able to discern the interaction various biological mechanisms with recurrent ischemic stroke in ICAD. Incorporation of perfusion imaging will disclose the role of hemodynamics and collateral flow at various timepoints. Vessel wall imaging studies will reveal how such atherosclerotic plaques change over time, while illustrating which imaging features such as enhancement or lipid-laden core are most relevant in clinical outcomes. The RISER study, including both asymptomatic and symptomatic patients with ICAD will explore such changes over 18 months, correlating imaging features with PCSK9 inhibitor treatment^1^.

Future ICAD studies are poised to leverage the extensive list of variables and availability of such data in routine clinical practice. Randomized, controlled trials should account for these individual-specific characteristics and clinical aspects when analyzing impact of the investigational treatment. Observational studies should ideally capture real-world data that is multidimensional, including imaging and diagnostics that measure underlying pathophysiology and physiologic response to treatments commonly employed for secondary stroke prevention in ICAD. Longitudinal or serial evaluation of patients will be essential to discern long-term clinical and imaging outcomes. ICAD is an ideal prototype for establishing precision medicine, capitalizing on the myriad variables commonly encountered.

## Author Contributions

DL conceived and designed the manuscript, analyzed and interpreted the data, handled funding and supervision, drafted the manuscript, and made critical revision of the manuscript for important intellectual content.

## Conflict of Interest

The author declares that the research was conducted in the absence of any commercial or financial relationships that could be construed as a potential conflict of interest.

## Footnotes

^1^Available online at: https://clinicaltrials.gov/ct2/show/NCT04573777
